# The Circulation

**Published:** 1869-10

**Authors:** N. M. Burkholder


### ARTICLE III.

### *The Circulation.*

By N. M. Burkholder, D.D S.

*Ccontinued.*

In proof of the position, we may further say that the circulation in any part greatly depends upon the activity of the functional changes taking place in it; that exercise of a part increase the changes in that part, and hence the circulation through it is more energetic.

The venous system arises, as before stated, out of a union of the capillaries as the arteries sub divide to form them, and conveys the impoverished blood back to the heart, before reaching which organ a fresh supply of nutrient material, both from the products of digestion and the recrementitial matter of the old tissues is poured into it. All the while the various glands are at work secreting from the blood what is particularly proper to themselves, or eliminating through the great waste gates of the system those properties which are in-nutritious and poisonous. Muscular action very much influences the rapidity of the venous circulation, owing to the compression of the vessels by their position and the valves preventing a reflux.

In the action of the heart, the contraction of the ventricles is synchronous as also that of the auricles,but the contraction of the auricles is synchronous with the dilitation of the ventricles and *vice versa*. The contraction of the heart is called the systole, the dilitation, the diastole. The rate of velocity of the circulation in different animals, bears a relation to the energy of respiration and this energy is governed by the activity of their functions, especially those of the nervous and muscular systems.

The action of the propulsion of the blood by the systole of the ventricles, produces the Pulse, of which we shall speak by and by. The sound or impulse of the heart is also felt to correspond with their contraction and dilitation and is due no doubt to the closure of the valves, the long sluggish sound to the tri-cuspid and mitral and the short sharper sound to the semi lunar. The walls of the left ventricle are thicker than those of the right, and the contractile power is greater just in proportion as the purpose to be answered is different. Each of the four cavities hold about two ounces of fluid. The whole quantity of blood appears generally to be about one-eighth the entire weight of the body, and the round of the circulation is made in from 40 to 60 seconds.

The number of contractions of this great organ in a given time, is subject to great variations, even in a state of health, from divers causes, governed by age, sex, exercise, state of mind, time of day, &c., &c. The infant's pulse beats 130 to 140 per minute, at adult age probably not over 70 or 80, and at as low as 50 to 65 in the decline of life. The female pulse is somewhat faster. As before said all muscular exertion sensibly increases the heart's action. In standing the pulse will generally range from six to ten beats faster per minute than when sitting, and perhaps four or five beats faster sitting than when in the recumbent position.

The mental emotions have a very powerful influence over the action of the heart, as all know.

We have so far failed to speak of the fœtal circulation. The blood of the fœtus is oxygenated in the placenta, its borrowed lungs, into which the vessels for its passage out, the umbilical arteries, enter and ramify, ending in what is known as placental tufts, very similar in their office to the rootlets of plants; these dip into the blood of the mother.

The return vessel, the umbilical vein, (and these vessels constitute the umbilical cord) enters the abdomen at the umbilicus, passes directly to the liver, gives a branch to the right and left lobe each, whilst a third and large branch, the ductus venosus, passes directly across to the inferior vena cava and being mingled with this ascending current, enters the right auricle and guided by a valve, the eustachian, passes directly through the "foramen ovale" into the left auricle without becoming intermixed with the current of the superior vena cava; from the left auricle it passes into the left ventricle and is thence impelled by its systole through the aorta, chiefly to the head and upper extremities and returning by the veins, is received into the right auricle and ventricle, from which latter it is thrown out through a vessel called the "ductus arteriosus," which takes its origin from the bifurcation of the pulmonary artery and opens into the aorta below the curve, which prevents its return to the head. It passes down the aorta along with that portion of aerated blood which the head &c., did not require of each systolic action of the left ventricle, to feed the lower extremities and to develope the organs in that region, and, in part, to be returned to the placenta for repairs, through the hypogastric arteries which emerging from the umbilicus, become the umbilical arteries, and together with the umbilical vein constitute the umbilical cord.

We can now understand why at birth, the brain is so much larger in proportion to other parts of the body, it receiving the best blood all the while, the lower extremities being compelled to *put up* with the crumbs. Soon after birth, however, contraction of this duct takes place, it degenerates into a fibrous cord, as does the ductus venosus, becoming the round ligament of the liver; the same fate belongs to the hypogastric arteries. The lungs having become developed just before birth and ready to perform their office, the respiratory apparatus at birth begins to act, in conformity with laws regulating it, the blood ceases to pass through the ductus arteriosus, but divides right and left and enters the lungs, the foramen ovale gradually closes by the development of a membrane from its margin, and the child becomes, physically, an independent being.

The action of the heart is supposed by some physiologists to be caused by a regular supply of nervous influence from the cerebro-spinal system, and this idea was seemingly supported from the fact that when the brain and spinal cord are removed, or when large portions of them are suddenly destroyed, the heart's action is stopped, but the shock to the whole nervous system may be the cause of such result, for we have instances of the removal of the entire brain gradually, with no such results. Another proof against this is furnished by anencephalus fœtuses.

Carbonic acid has been by some writers assigned as the special stimulus or cause of its action, arguing upon the fact that if the breath is held the heart's action is very much increased. This, however, is doubtless due to a reflex action of the nerves calling upon the heart to send more oxygen to the tissues, to the brain, which feels its loss ; the heart responds and thus its action is increased—in other words, a conservative effort of nature to rid the system of the poison. Others believe there is no special nervous influence upon which its movements are dependent, beyond that of simple nervous irritability; and that the muscular heart is possessed of such irritability, that its proper stimulus, the blood, excites it continually to action. Opposed to this view we have the fact that the pulsation can be detected in the embryo, when the heart is yet but a simple mass of cells.

We may fairly conclude then that little or nothing is known of the prime cause of the heart's action.

Violent impression may suddenly suspend its action,—we have an exhibition of it in syncope.

After the blood passes in this jet-like manner into the arteries, it would proceed in the same manner, were it not for the equalization of its movement by the elastic coat of the arterial walls. The muscular coat regulates its calibre, and at a distance from the heart, the only sign we have of its jet-like action is a greater and lesser rapidity of the flow, causing a rise and fall of the current, and is known as the *pulse.* In other words, the pulse is a statement of the force of contraction of the left ventricle, reflected from the heart outwards by molecular transmission ; an exact report of the movement and condition of the heart and of the condition of the arteries themselves.

The ramifications of the arteries communicate freely with each other and those of the veins; if possible more freely by inosculation. We see the wisdom in this provision of collateral circulation when we obstruct a main trunk by ligation.

The pulse takes a conspicuous position in all diagnoses, and the importance of perfect familiarity with its *host* of variations and indications, is apparent to every student of medicine, if he would attain, truly, the title of a *successful practitioner*

The human circulation with all its various phenomena is, truly, a subject of extraordinary interest, and although, in this article, I have been only able to give a brief description of these phenomena, yet the appreciating and inquiring mind cannot fail to take up the subject and prosecute it to a satisfactory termination.

An exceedingly and *intensely interesting* study to him that would master disease, that would diagnose truly so many of its forms which are now certainly obscure, as regards their essential nature, is—the blood, the changes which take place in it in disease and in health.

Upon this subject the scientific world is yet in its infancy, and until better understood, until the immediate changes caused in the ultimate elements of the blood by foreign matters, whether solid or gaseous, are explained, until, in its poisoned condition, its altered relations to the tissues is more plainly comprehended, how can we expect to cure disease ?

